# An iridescent film of porous anodic aluminum oxide with alternatingly electrodeposited Cu and SiO_2_ nanoparticles

**DOI:** 10.3762/bjnano.10.73

**Published:** 2019-03-19

**Authors:** Menglei Chang, Huawen Hu, Haiyan Quan, Hongyang Wei, Zhangyi Xiong, Jiacong Lu, Pin Luo, Yaoheng Liang, Jianzhen Ou, Dongchu Chen

**Affiliations:** 1 College of Materials Science and Energy Engineering, Foshan University, Jiangwan First Road, Chancheng, Foshan, Guangdong, China; 2 School of Engineering, RMIT University, Melbourne, VIC, Australia

**Keywords:** aluminum alloys, anodic aluminum oxidation, interference-enabled color production, rainbow effect, structural color

## Abstract

The structurally colored surface of anodic aluminum oxide (AAO) is highly useful for decoration and anti-counterfeiting applications, which are of significance for both scientific and industrial communities. This study presents the first demonstration of the fabrication of an iridescent film of porous AAO on an industrial aluminum alloy substrate, with alternatingly electrodeposited Cu and SiO_2_ nanoparticles (NPs). A rainbow effect was effectively obtained for the optimized sample with appropriate alternating electrodeposition times. The structure and optical properties of a series of the electrodeposited AAO-based thin film were investigated. The Cu and SiO_2_ NPs were found to be uniformly deposited into the porous structure of the AAO film, and the alternating electrodeposition repeating twice led to the formation of the optimal AAO-based thin film that exhibited a rainbow effect and superior anti-corrosion performance.

## Introduction

Due to the low cost, high mechanical strength and ductility, and well-developed production procedures, aluminum alloys have been extensively used as nonferrous structural materials [1–12]. Aluminum alloys are generally prepared by doping aluminum with other elements at a low content [13]. Anodic aluminum oxidation processing and electrodeposition treatment can allow the aluminum alloy to bear different structural colors, providing exciting opportunities for bringing such materials to the fields of decorative materials [14–17]. However, only a limited number of colors has been produced by the traditional coloration techniques [14,16,18–19]. To widen the spectrum of colors, many researchers turn to mimic the structural color from nature, which is expected as the origin for the artificial creation of multiple and stable colors existing on the surface of aluminum alloys [15,20]. Furthermore, their own characteristics of the structural colors are also expected to enrich the aluminum alloys with vivid optical properties [21].

Structural colors are generated by light diffraction, interference and scattering [20], completely different from pigment colors involving the selective absorption of a certain light waves and the reflection of the complementary light waves. Structural colors do not absorb light, implying that the light intensity will not decrease, and significantly, a local iridescent phenomenon appears as a result of light diffraction and reflection. This rainbow effect of structural colors refers to that different colors are displayed with a change of the viewing angle [16,22]. By contrast, no rainbow effect occurs in the pigment colors. The artificial structural color is inspired from nature, e.g., the bright tail of the peacock feathers, the mixed cyan and green shell of the Coleoptera beetles, and the wings of butterflies [15]. In comparison with pigment colors, structural colors are much more stable, as a color change can only take place when the physical structure is varied [23].

Two types of strategies have been employed to produce structural colors, one is based on self-assembly methods [24], and the other is based on electrodeposition [18]. The former involves the simultaneous assembly of the basic structural units such as molecules, nanomaterials, and the materials on the microscale or even larger scales to form an ordered structure. During the self-assembly process, the basic structural units organize or aggregate, in a simultaneous way, into a stable structure possessing a regular geometric appearance. In contrast, electrodeposition involves the nucleation at an electrode surface under the action of an electric field [25]. For example, a high-purity aluminum foil was directly used as a template, on which anodic aluminum oxide (AAO) films with different thicknesses were generated by anodization for different durations. Subsequently, the electrodeposition of Co and Cu were performed. Under irradiation of natural light perpendicular to the surface, different colors (including purple, blue, blue-green, green, and yellow) appeared in the Co/AAO films depending on the anodization duration, and another set of colors (including purple, indigo, blue, blue-green, and green) also appeared in the Cu/AAO films depending on the oxidation time. This colorful AAO composite film with the electrodeposited metal was a result of an increase in the effective refractive index and hence reduction of the reflection of the aluminum substrate [25]. The saturation of the structural colors of the metal-deposited AAO composite film was largely enhanced [25–27]. An electrostatic self-assembly technique was also employed to produce large-area, ordered interference-enabled colored films with uniform structural colors on the surface of inorganic nanoparticles (NPs) that had been prepared to bear surface charges [28]. On the substrates of quartz glass, PET and PP, twenty cycles of the assembly of a SiO_2_ film led to the formation of dark red, orange-yellow, and lake-green films, respectively. With a change in the particle size of SiO_2_, the PET substrate after being exposed to twenty cycles of SiO_2_ film deposition exhibited a color variation from blue over magenta to green. Varying the cycles of the deposition of 50 nm SiO_2_ film, color changes were demonstrated with the incident light angle, e.g., from cyan to blue, from orange-red to yellow, from blue-green to blue-purple, and from magenta to dark green. The SiO_2_/PET film was also applied to the surface of textile fibers, yielding structural colors [29–31]. Using a one-step oxidation method in phosphoric acid solution, AAO/Al was firstly prepared, onto which a non-magnetic Ag@AAO composite film was further fabricated by an alternating electrodeposition technique. It was found that under incident light at 0°, the color of the Ag/AAO film changed with the electrodeposition time, including purple, blue, green, yellow, pink, and red. Varying the incident light angle, different colors were exhibited including dark yellow, dark green, dark blue, and light purple. A picture was also created on an organic coating that was previously applied onto the Ag/AAO film, and different patterns could be generated with the variation of the incident light angle, satisfying the requirements for optical anti-counterfeiting applications [32]. A self-made electrophoresis-based deposition device was also adopted to deposit negatively charged PS spheres onto the surface of a carbon fiber using a stainless steel tube and a carbon fiber as the anode and cathode under the action of a circular electric field, respectively, resulting in a cylindrical fibrous structure. The control over the electrodeposition voltage and time allowed for the fabrication of fibers with different thicknesses, and the resulting fibers exhibited structural colors of blue, green, and red when the PS spheres with a diameter of 185, 230 and 290 nm, respectively, were employed [33–35]. A natural sedimentation method was also used to prepare a structurally colored SiO_2_ photonic crystal film. Changing the incident light angles led to a variation of the structural color from red to blue-purple, and the SiO_2_ particle size was also found to have an influence on the film color [14]. Furthermore, an AAO template was firstly prepared in an electrolyte with an alkaline silica gel and phosphate, onto which a layer of an Au film was deposited via sputtering, yielding a colorful filter material. Different structural colors could be obtained via changing the anodization time [17].

In this context, instead of using high-purity aluminum foils and titanium foils as the substrate for the anodization treatment, which have been widely explored [25,32,36–37], we employed an industrial aluminum alloy as substrate to first generate porous AAO films and subsequently investigated the structural color exhibited in the AAO films after alternating electrodeposition of Cu NPs and SiO_2_ NPs with high and low refractive indexes, respectively. The NPs grew in the porous AAO film in a confined manner. The large difference between the refractive indexes of the Cu NPs and the SiO_2_ NPs could result in the generation of vivid colors. This study presents the first demonstration of tailoring the structural coloring of AAO film-decorated industrial aluminum alloy plates by controlling the times of the alternating electrodeposition of Cu and SiO_2_ NPs. Interference-related colors were achieved and the rainbow effect of the structural color was also observed. The study presented here will stimulate the advancements of the utilization of structural colors with high stabilities for a wide range of applications such as colorful case shells of electronic devices, automobile bodies, and anti-counterfeiting labels.

## Experimental

### Materials

The 6063 aluminum alloy was adopted as substrate. Tetraethoxysilane and sodium dodecyl sulfate (SDS, used as a surfactant) were of analytical reagent (AR) grade and obtained from Fuchen Chemical Reagent Factory. Potassium nitrate (AR) was supplied by Guangzhou Chemical Reagent Factory, and all the other reagents were AR grade and purchased from Guangdong Guanghua Sci-Tech Co., Ltd.

### Anodization and electrodeposition-based structural coloring of the aluminum alloy

#### Pretreatment

For removing oily contaminants and dirt, the 6063 aluminum alloy sample was first washed in an alkaline solution for 3–4 min, and then washed with deionized (DI). Afterward, it was put into an acidic eluent for the acid-based washing for 2–3 min and then thoroughly washed with DI water. Before stored for later use, the sample was blow-dried. The alkaline solution was composed of NaOH (40.0 g), SDS (1.0 g) and DI water (1.0 L), while the acidic solution consisted of sulfuric acid solution (40%) and nitric acid solution (10%).

#### Anodization

The pretreated sample was placed in a sulfuric acid solution (117 g/L), and the DC electrical power supply (KXN-305D) was switched on. The anodization was conducted for 30 min at 0–6 °C and an oxidation current of 1.2 A. During the oxidation, the sample was kept parallel to the two cathodes, with equal distances between sample and each cathode.

#### Pore-enlarging treatment with phosphoric acid

After anodization, the sample was put into a phosphoric acid solution (5%) and allowed to stand for 12 min, and then DI water was employed to remove the excess phosphoric acid solution, followed by blow-drying.

#### Galvanic deposition of Cu NPs

In a CuSO_2_ solution (60 g/L), the anodically oxidized sample was colorized using an electrical supply (EOECD-30A) with a constant voltage of 15 V for a deposition time of 35 s. During the electrodeposition process, the sample was kept parallel to the electrodes and at equal distances between them. The sample was taken out of the electrolyte and then blow-dried before storage for later use.

#### Galvanic deposition of SiO_2_ NPs

The sample with the electrodeposited Cu was put into a SiO_2_ deposition liquid, and the power supply (EOECD-30A) started with a constant voltage of 3 V for 35 s deposition. The sample was kept parallel to the electrode, and the distance between the sample and electrodes was kept equal during the deposition process. After the deposition, the sample was removed, washed with DI water to get rid of the SiO_2_ deposition liquid, blow-dried with a hair dryer, and finally put into a sealed pocket for later use. The SiO_2_ deposition liquid was prepared by mechanically mixing potassium nitrate (10.11 g), DI water (500 mL), absolute ethyl alcohol (500 mL), adding tetraethoxysilane (50 mL) after the pH value was adjusted to 3. The flow chart for the stepwise galvanic deposition is presented in Table 1.

**Table 1:** Alternating electrodeposition of Cu and SiO_2_ for the preparation of different samples.

sample	deposition order

S1	Cu
S2	Cu→SiO_2_
S3	Cu→SiO_2_→Cu→SiO_2_
S4	Cu→SiO_2_→Cu→SiO_2_→Cu→SiO_2_

#### Hole sealing by hot water

After the alternating electrodeposition, the sample was immediately put into pre-boiled distilled water, and allowed to stand for 40 min. It was subsequently removed and blow-dried before placed into a sealed pocket for later use. The purpose of sealing the pores with hot water was to close the pores in the anodic oxide film and hence to avoid impurities entering the film.

#### Electrochemical properties

Electrochemical impedance testing was carried out by applying a small-amplitude AC voltage to the system and measuring the ratio of the signal voltage to the current (this ratio was defined as the system impedance) with the change of the sinusoidal-wave frequency, or the variation of the phase angle of the impedance with the change in frequency. Nyquist and Bode diagrams can be obtained by the electrochemical impedance measurements. The interfacial impedance of the sample was estimated on the basis of the analysis described above, and the corrosion resistance performance was evaluated in more detail. Furthermore, the electrical polarization process of the sample in the test solution was studied by analyzing the Nyquist and Bode diagrams.

The test sample was immobilized onto a PVC tube with epoxy resin, and then naturally dried for 12 h. After that, a NaCl solution (3.5%) was poured into the PVC tube, and the level of the NaCl solution was in the range of a half to two-thirds of the tube volume. The exposed area of the PVC tube was approximately 5.7 cm^2^. After being allowed to stand for 24 h, the sample was exposed to the electrochemical impedance measurements. A three-electrode system was adopted for the measurements, and a Pt wire and a saturated calomel electrode (SCE) were employed as the auxiliary electrode and reference electrode, respectively. Because such an electrochemical impedance measurement is sensitive to the outside interferences, the workstation was not permitted to be disrupted during the measurement, and electronic devices such as mobile phones were placed far away from the experiment.

The polarization test was divided into constant-potential scanning and constant-current scanning, while the former was divided into electrostatic potential scanning and dynamic potential scanning. The dynamic potential scanning was mainly implemented because of its advantages of automatic mapping and controllable scanning speed. The potentiodynamic sweep was performed by controlling the electrode potential in a manner of continuously changing (scanning) at a slower speed, and the instantaneous current value at the corresponding potential was measured. The instantaneous current was plotted as a function of the corresponding electrode potential to obtain the entire polarization curve. Since the potential of the electrode applied by the potentiostatic potential was sufficient to destroy the barrier layer of the sample to be tested, the potentiodynamic scanning must be performed after the electrical impedance test had been completed. The potentiodynamic sweep proceeded from −1.5 V to 1.5 V; the scanning speed, sampling interval, and frequency were set as 1.5 mV/s, 0.5 s, and 2 Hz, respectively.

#### Characterizations

A DC power supply (KXN-305D) was employed to conduct the alternating electrodeposition for achieving the structural coloring. The power supply (EOECD-30A) was adopted for the anodization processing of 6063 aluminum alloy samples. During the anodization, a conversion-based refrigerator (BC/BD-143), a non-contact infrared thermometer (AR842A+), and an electrically heated thermostatic water bath were used to strictly control the temperature. The electrochemical measurements were carried out using an electrochemical workstation (CS-310) in a three-electrode system, where the platinum electrode and saturated calomel electrode (SCE) worked as the counter and reference electrodes, respectively. The scanning range of the potentiodynamic polarization curve was set from −1.0 to +0.5 V, with a scanning rate and a sampling interval of 1 mV/s and 1 s, respectively. The range of the AC impedance test rate and the AC amplitude of the sinusoidal wave were set from 10^−2^ to 10^5^ Hz and to 10 mV, respectively. The wide-band responses at the frequencies above 10 Hz and below 10 Hz were 470 pF and 2.2 nF, respectively. The parameters related to filter and earthing modes were set as 470 nF and field, respectively. The prepared samples were observed using a TM3030 scanning electron microscope (SEM, Hitachi). The absorbance and emissivity properties were measured using an ultraviolet–visible–near infrared (UV–vis–NIR) spectrophotometer (UV-4100, Hitachi, Japan). Microstructure observation and phase-composition analyses were performed using a TD-3500 X-ray diffraction (XRD) instrument. For measuring the thickness of the thin films formed on the sample, the work probe of a cladding thickness gauge (MINITEST 600) was placed on the specimen surface after the anodization, and the thickness could be directly read on the gauge. We arbitrarily selected 7–8 positions that were distributed all over the film surface in order to measure the average thickness of the film.

To scientifically describe the color of the sample, CIERGB, CIEXYZ and CIELAB models were successively established by International Lighting Commission, and CIELAB is considered as the most complete color model to describe the color observable by the naked eye. CIELAB (CIE1976*) consists of three channels, i.e., the *L*, *a* and *b* channels that represent brightness, red and green, and yellow and blue, respectively (where the larger the *a* value, the closer to red is the color, and the inverse leads to green color; the larger the *b* value, the closer to yellow is the color, and inverse results in blue color. The color difference between two samples can be evaluated according to the established color difference formula.

The change of the incident light angle led us to observe the rainbow effect of the structurally colored film. In this study, the most obvious rainbow effect of the structure was regarded as the best sample, that is, the sample bearing the maximum color difference under varying angles of incident light. According to the spectral photometric method in the standard GB/T 3979-2008, the object colors of the sample under 2° incident light and 10° incident light were measured. The chromatic aberration of each sample at two different angles of incident light was calculated, and the optimal group had the most chromatic aberration. The chromatic difference formula (E*) is given below:

[1] $$E^{*}={\left[{\left(L_{1}{}^{*}-L_{2}{}^{*}\right)}^{2}+{\left(a_{1}{}^{*}-a_{2}{}^{*}\right)}^{2}+{\left(b_{1}{}^{*}-b_{2}{}^{*}\right)}^{2}\right]}^{1/2}.$$

## Results and Discussion

**Figure 1:**
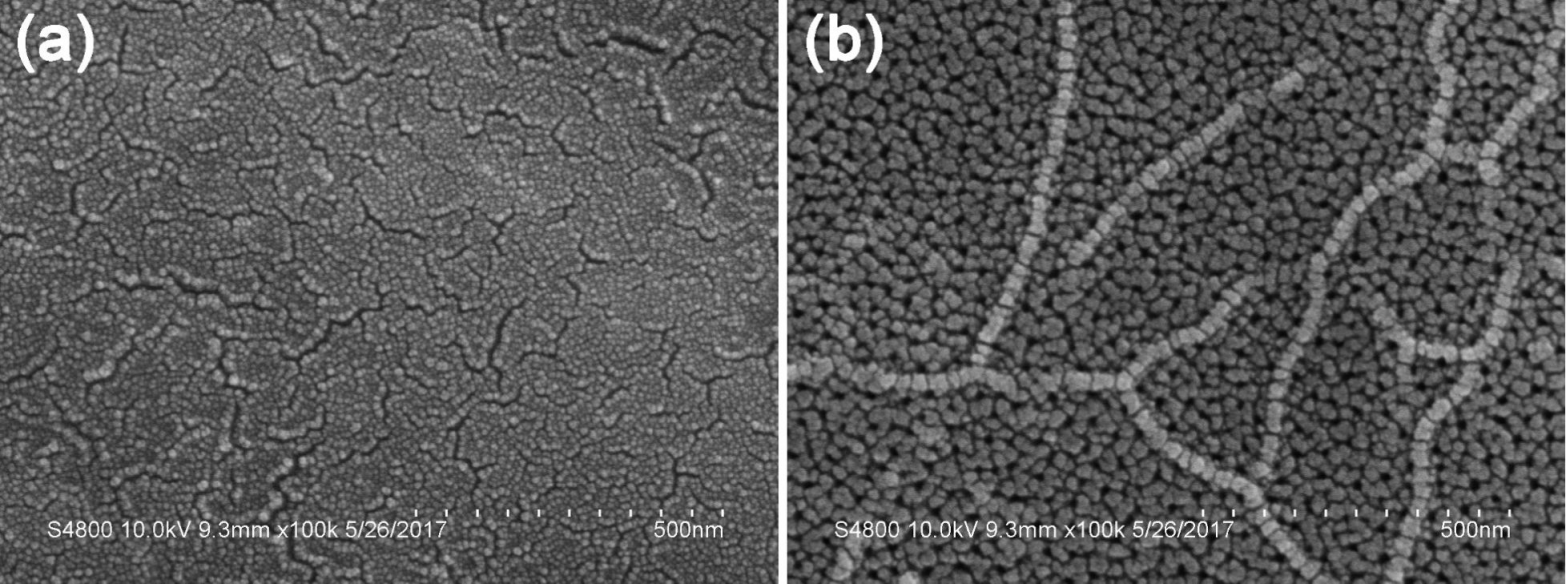
a,b) SEM images of the surface of the aluminum alloy before (a) and after (b) anodization.

The oxidation was performed for 30 min in the oxidizing solution at 0–6 °C with an oxidation current of 1.2 A. The thickness of the resulting anodized film was maintained at 16–18 µm. Film thickness tests were performed on the samples deposited with Cu and SiO_2_ NPs, and no thickness growth could be detected after the alternating electrodeposition. This is most likely due to that both Cu and SiO_2_ were deposited into the pores of the porous AAO film, and thus the film thickness was consistent with the initial one. Figure 1a shows the microscopic morphology of the bare aluminum alloy before anodization, and a smooth surface of the aluminum alloy, without any big cracks, can be seen. In contrast, after anodization, the aluminum alloy becomes more porous, with a large number of holes on the AAO film (Figure 1b), revealing that the anodization treatment leads to the generation of a porous aluminum oxide film on the surface of the aluminum alloy substrate. Considering the limited size of the pores in the AAO film, the pore-enlarging treatment is needed to facilitate the electrodeposition process.

**Figure 2:**
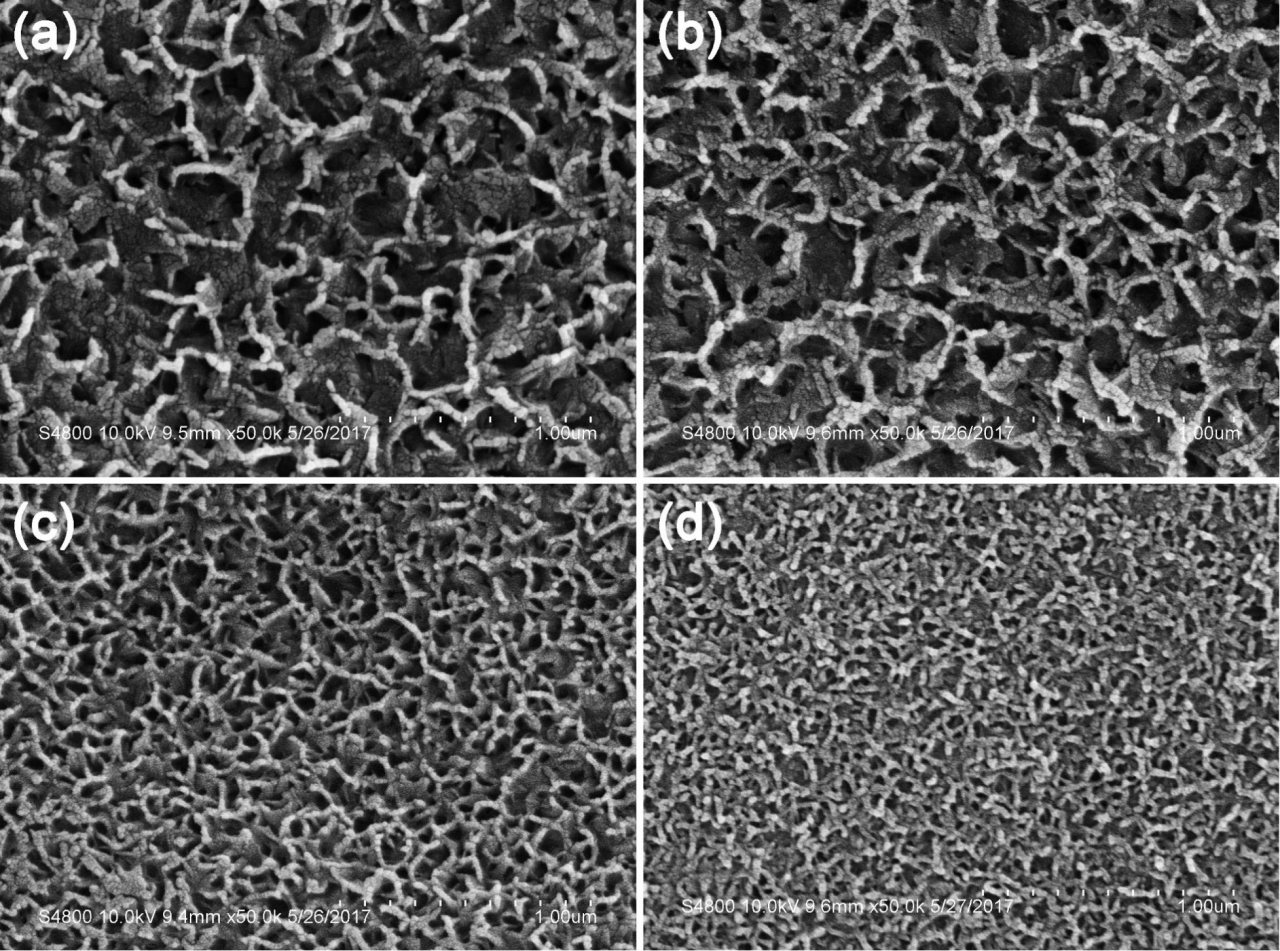
a–d) SEM images of the prepared samples: S1 (a), S2 (b), S3 (c), and S4 (d).

From Figure 2a, pores in the AAO film can be clearly observed for the sample S1, which also indicates that only one step of electrodeposition of Cu NPs into the holes does not significantly alter the structure and morphology of the AAO film. After deposition of SiO_2_, the morphology is greatly changed for sample S2, and it is more difficult to notice the distribution of the AAO film in Figure 2b. In addition to the Cu NPs deposited in the pores of the AAO film, SiO_2_ NPs can also be observed within the pores. The electrodeposition of an additional Cu layer resulted in the sample S3 with reduced visibility of the AAO film pores (Figure 2c). Most of the pores are filled after the repeated electrodeposition, and the entire surface appears even, without observable cracks. The SEM image of sample S4 is presented in Figure 2d, and the porous AAO film becomes more densely packed with electrodeposition layers that are homogeneously distributed over the entire surface in a crack-free way. It can be noted from Figure 2 that the electrodeposited layers of Cu and SiO_2_ NPs were arranged in an ordered manner, which might be the cause of the iridescence.

**Figure 3:**
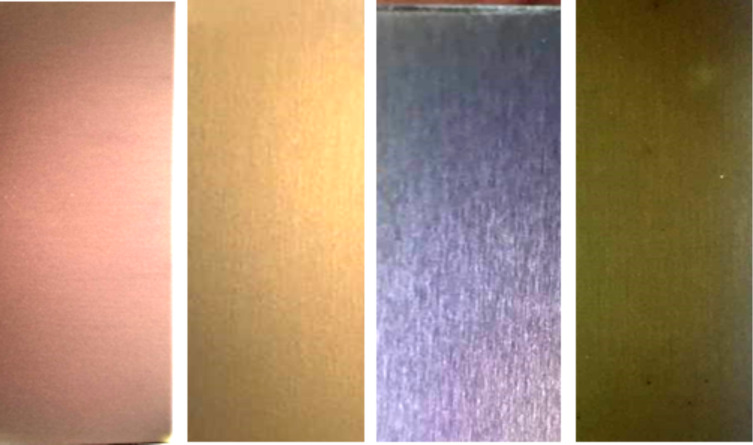
Digital images showing the colors of the prepared samples (from left to right: S1, S2, S3, and S4 ); the sample surface is perpendicular to the incident light.

The colors of the various prepared samples are presented in Figure 3, with the incident light perpendicular to the sample surface. At the same angle of the incident light, the electrodeposited films exhibit colors progressing from purple-red, light brown, brown, purple, red and to brown-green with increasing numbers of electrodeposition cycles. Upon the change of the angle of the incident light from 0° to 30°, the rainbow effect appears only for the sample S3, as shown in Supporting Information File 1, Figure S1. The structural color changes from light purple to dark purple with the variation of the incident light angle from 0 to 30°.

**Figure 4:**
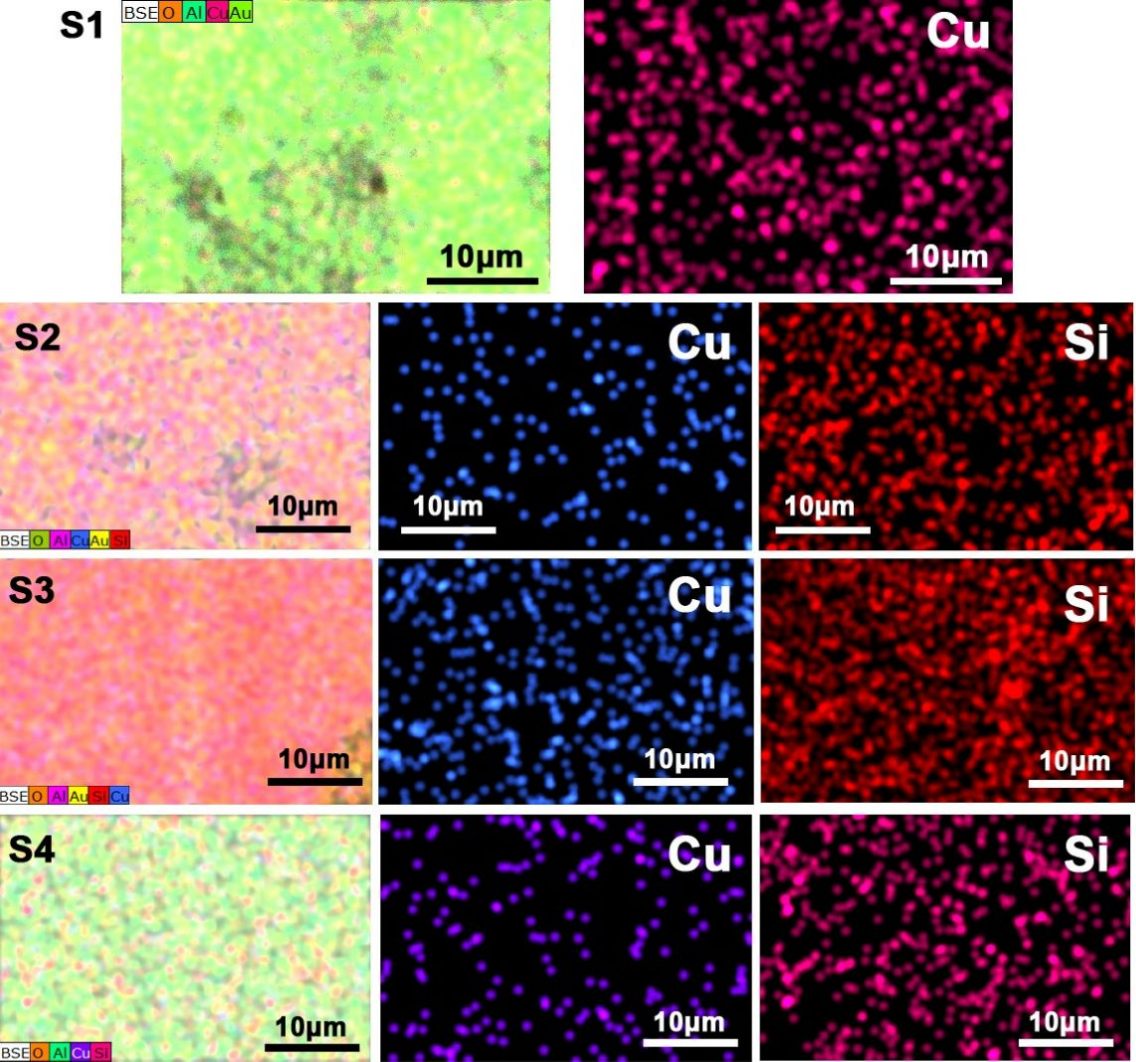
Elemental mapping images of the prepared S1, S2, S3, and S4 samples (progressing from top to bottom); the leftmost images correspond to the mapping images of full elements.

From the elemental mapping images of the S1 sample shown in Figure 4a, the sample includes the elements of Al, O, Cu and Au. The appearance of Au is due to the sputtering of the surface with Au (to enhance the electrical conductivity of the sample) before scanning. Cu can be seen as numerous particles at the nanoscale homogeneously distributed over the entire surface. In sample S2 (Figure 4b), Si can be observed all over the sample surface, revealing the uniform deposition of SiO_2_. The electrodeposited Cu and SiO_2_ are believed to be confined to the pores of the AAO film. Similarly, the S3 and S4 samples also exhibit a uniform distribution of Cu and SiO_2_ NPs.

**Figure 5:**
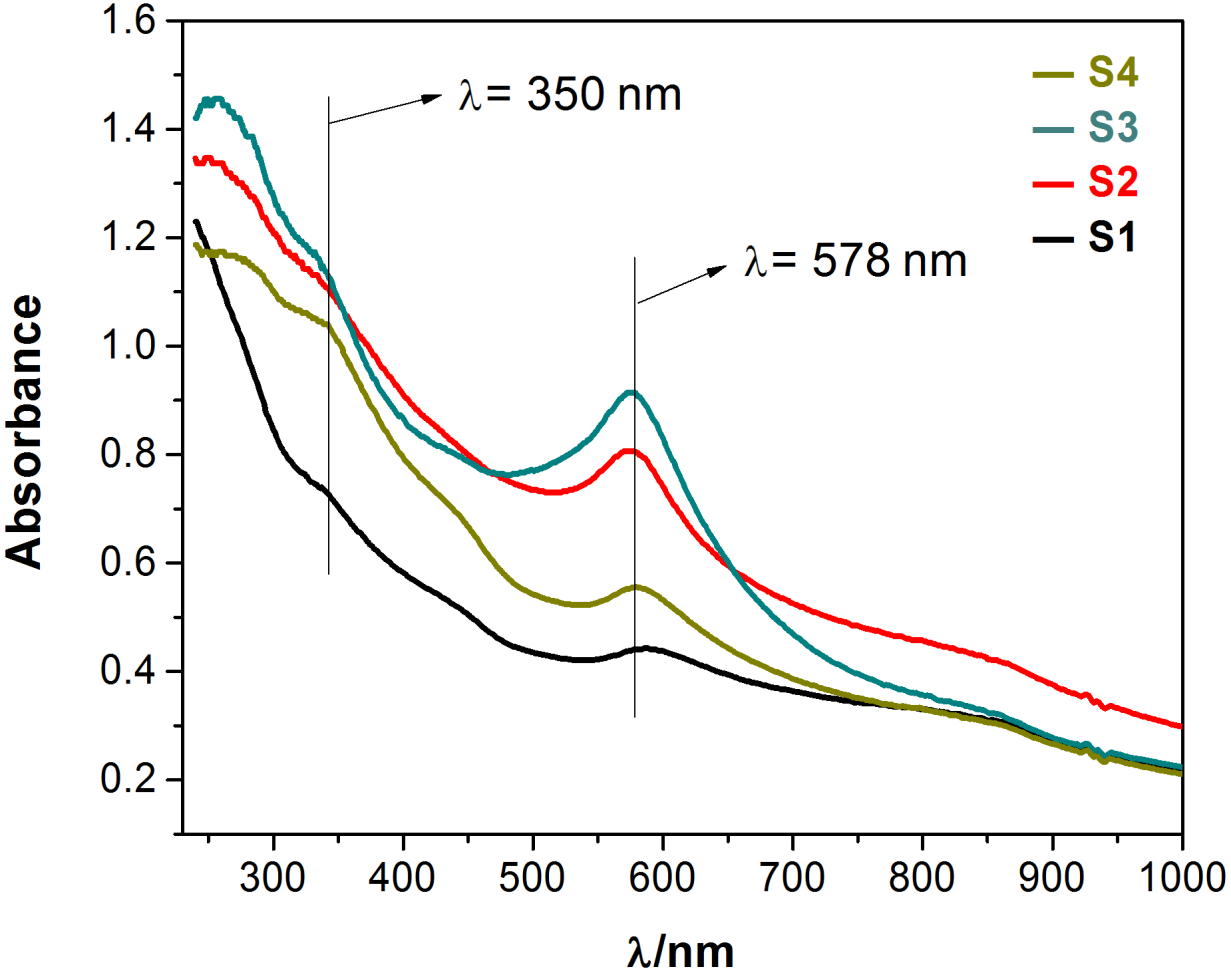
UV–vis–NIR absorption spectra of the various prepared samples.

In the UV–vis spectra (Figure 5), all samples exhibit a strong absorption at 578 nm and a relatively weak absorption at 350 nm, which indicates the existence of the Cu NPs. The UV–vis absorption of SiO_2_ is mainly in the ultraviolet and far ultraviolet. The absorption at 350 nm is a result of plasmonic resonance absorption from Cu, while the peak at 578 nm can be assigned to Cu NPs. The absorption intensities at 350 and 578 nm gradually increase with the increase of the electrodeposition times corresponding to the samples S1 to S3, but decrease again for sample S4.

To further validate the color difference of the samples, the chromatic difference was analyzed according to the spectrophotometric colorimetry in the standard GB/T3979-2008, and the values of L*, a*, and b* were estimated under incident light at angles of 2° and 10°. The rainbow effect is useful for anti-counterfeiting applications. The sample with the most obvious rainbow effect, that is the largest chromatic difference, can be regarded as optimal one. The values of L*, a*, b* and color difference (E*) are presented in Table 2 and Table 3. Among the prepared samples, sample S3 exhibits the largest value of E* (1.9038). As also proven in Supporting Information File 1, Figure S1, the change of the incident light angle from 0° to 30° results in a noticeable color variation of sample S3. By contrast, the color of the other sample samples is independent of the incident light angles.

**Table 2:** L*, a*, and b* values under different incident light angles.^a^

sample code	incident light at 2°			incident light at 10°		
	L*_1_	a*_1_	b*_1_	L*_2_	a*_2_	b*_2_

S1	57.2714	−2.4112	4.2059	57.2272	−2.6253	4.5684
S2	49.1042	2.2042	4.5211	49.0606	1.3158	4.8799
S3	47.0084	5.2818	−0.4565	47.1218	3.4356	−0.0056
S4	59.5853	−2.4937	11.2013	59.3624	−2.2635	11.5646

^a^All calculations were conducted in triplicate, with the uncertainties (u) of the above parameters calculated as the standard deviation, and the u values of all of the above parameters were found to be within 6 × 10^−4^.

**Table 3:** Color difference (E*).

sample	S1	S2	S3	S4

E*	0.4253	0.9591	1.9038	0.4844

The color results from interference in the film, and the thickness of the film must not be too large if to produce a color effect. This is because two waves of reflected light will be generated at the top and the bottom surface of the irradiated film. The occurrence of interference requires that the frequencies of the two reflected light waves keep the same, with the same vibrational direction. As a result, the film thickness should be sufficiently low to ensure that the two reflected waves have the same frequency and vibrational direction. When the thickness of the film is too large, there is a big difference in the optical path length, which is detrimental to the consistency of the frequency and vibrational direction. This explains why sample S4 exhibits less interference than sample S3.

The relationship among the interference wavelength of the multilayer film, and the refractive index, thickness and refraction angle of the double-layer film can be expressed by Equation 2, and the schematic diagram is presented in Supporting Information File 1, Figure S2.

[2] $$m\lambda =2\left(n_{1}d_{1}\cos\theta_{1}+n_{2}d_{2}\cos\theta_{2}\right),$$

where *n*_1_ and *n*_2_* r*epresent the refractive indexes of the film, θ_1_ and θ_2_ are the refraction angles, and *d*_1_ and *d*_2_ are the film thicknesses [29–31].

At a given thickness, the larger the number of the layers is, the larger is the difference in the refractive index, leading to stronger interference. Theoretically, the highest interference can be obtained when *n*_1_*d*_1_, *n*_2_*d*_2_, and λ/4 are equal to each other.

Progressing from S1 to S4, the color difference increases first and then decreases again; the former is attributed to the multilayer interference that makes the rainbow effect increasingly more obvious. In sample S4, the film thickness becomes too large reducing any interference effects.

**Figure 6:**
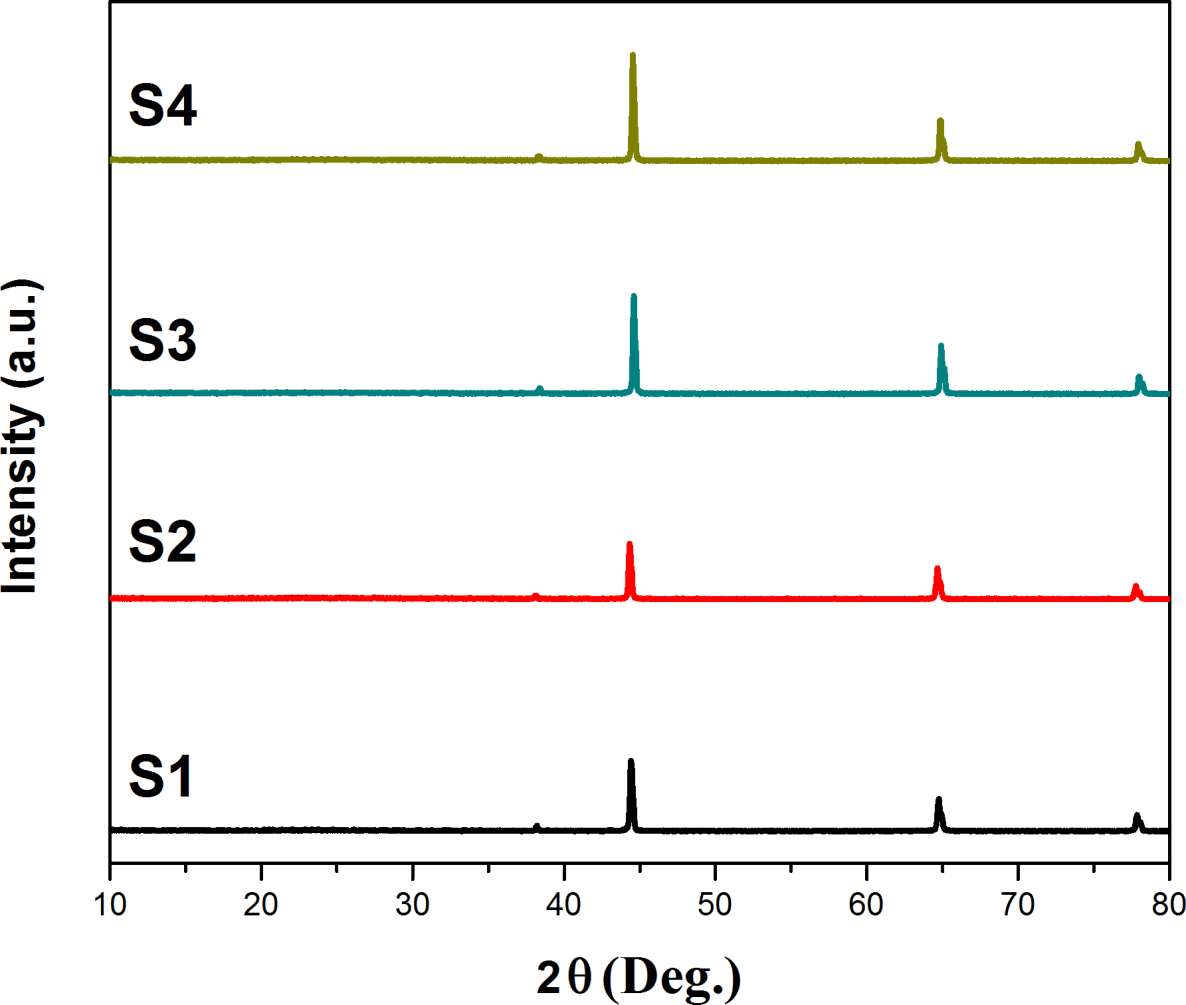
XRD patterns of the various prepared samples.

XRD patterns of the prepared samples are presented in Figure 6, and all of the samples exhibit similar diffraction patterns with peaks at 38.46°, 44.76°, 65.24°, and 68.31°, assigned to the diffraction planes of Al(111), Al(200), Al(220), Al(311), respectively. There is no diffraction that can be indexed to SiO_2_, which might because it is present in the film as an amorphous state. Also, no signal can be noted for Cu species, which is most likely due to the low content of Cu that is below the detection limit of the XRD equipment.

**Figure 7:**
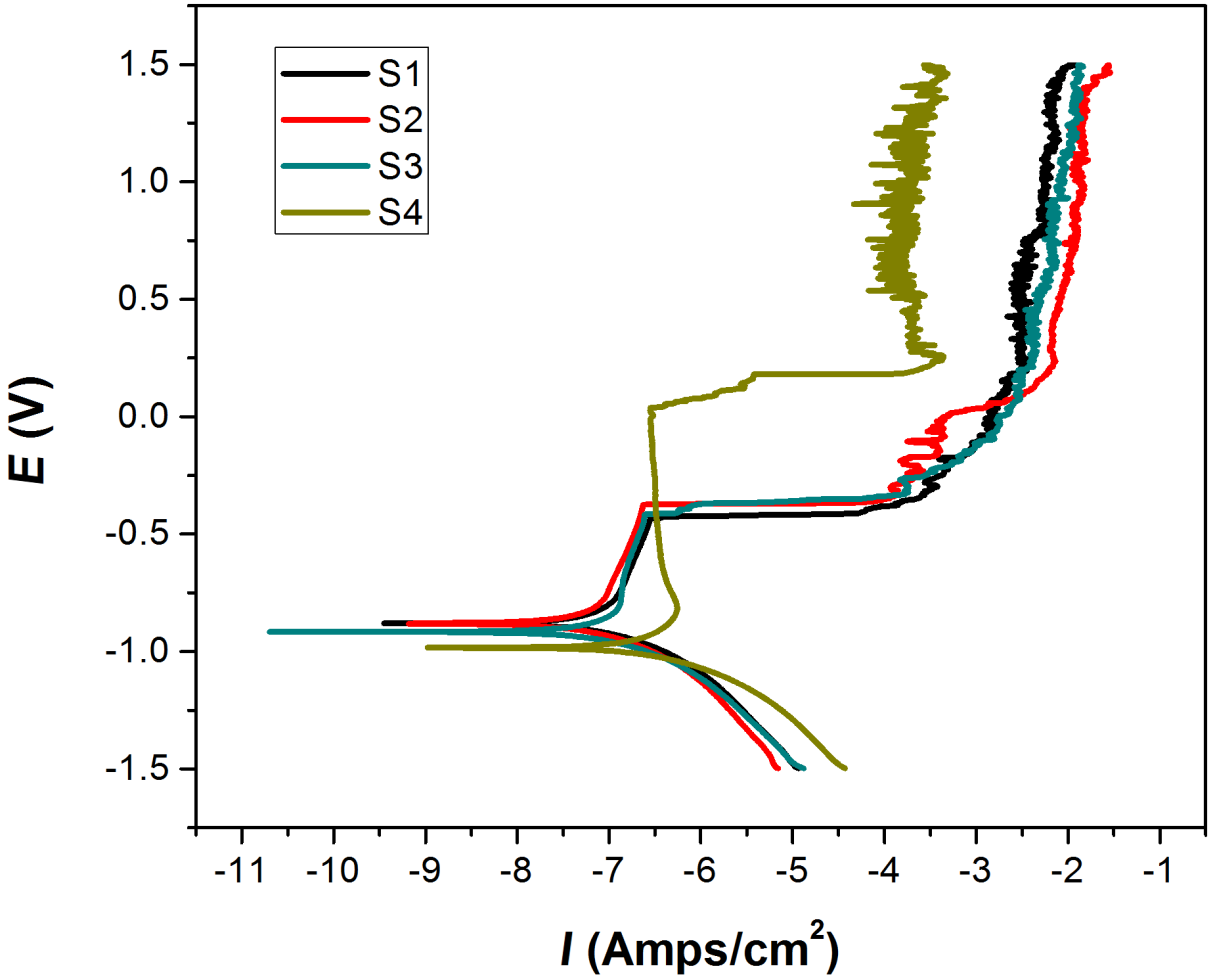
Electric polarization curve for the various prepared samples.

In the electrical polarization measurements, the self-corrosion current, *I*_corr_, and self-corrosion potential, *E*_corr_, are critical parameters to evaluate the corrosion resistance of materials, especially aluminum profiles (Figure 7 and Table 4). Generally, the smaller the value of *I*_corr_ is, the higher is the hole-sealing quality. Larger values of *E*_corr_ can be an indication of higher corrosion resistance. From the values of *I*_corr_, the best hole-sealing quality can be found in the sample S3, while the sample S2 can be regarded as the optimal one as far as *E*_corr_ is concerned. Nevertheless, the quality evaluation based on the values of *I*_corr_ is more widely employed, and therefore the results obtained via electric polarization measurements demonstrate that the sample S3 possesses the best quality in hole sealing.

**Table 4:** Polarization parameters of the prepared samples.^a^

sample code	polarization parameter				
	*Ba* (mV)	*Bc* (mV)	*I*_corr_ (A/cm^2^)	*E*_corr_ (V)	*R*_corr_ (Ω cm^2^)

S1	385.95	142.84	8.5422 × 10^−8^	−0.88067	0.0010047
S2	556.19	164.64	8.0341 × 10^−8^	−0.88038	0.00094498
S3	214.47	110.87	5.4434 × 10^−8^	−0.92124	0.00064026
S4	545.35	153.87	3.6714 × 10^−7^	−0.9837	0.0043183

^a^The polarization parameters were calculated based on the Tafel fitting of the curves presented in Figure 7, with the u(*Ba*) < 0.05, u(*Bc*) < 0.04, u(*I*_corr_) < 8 × 10^−12^, u(*E_corr_*) < 2 × 10^−4^, and u(*R*_corr_) < 3 × 10^−7^.

**Figure 8:**
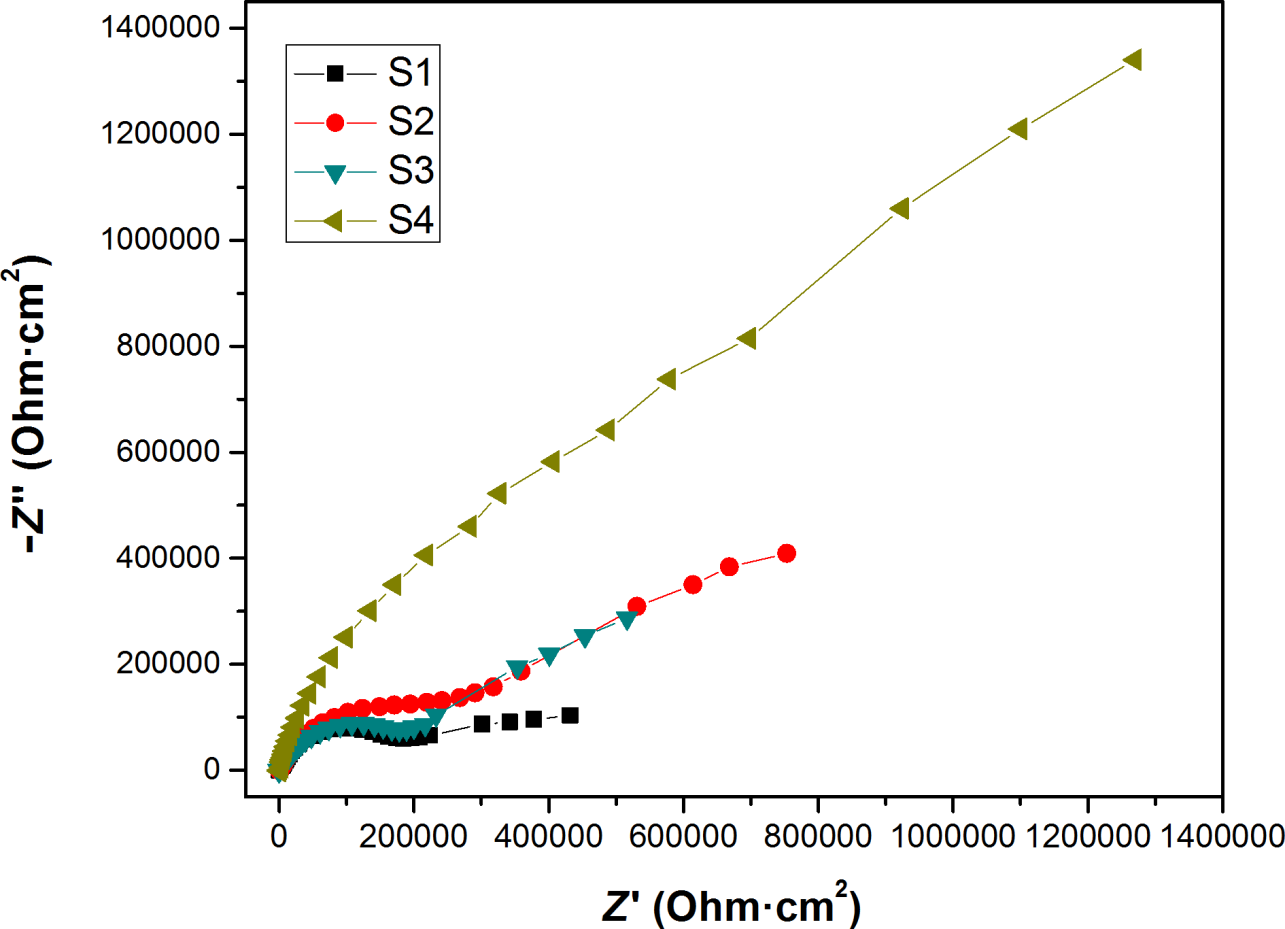
Nyquist diagram of the prepared samples.

**Figure 9:**
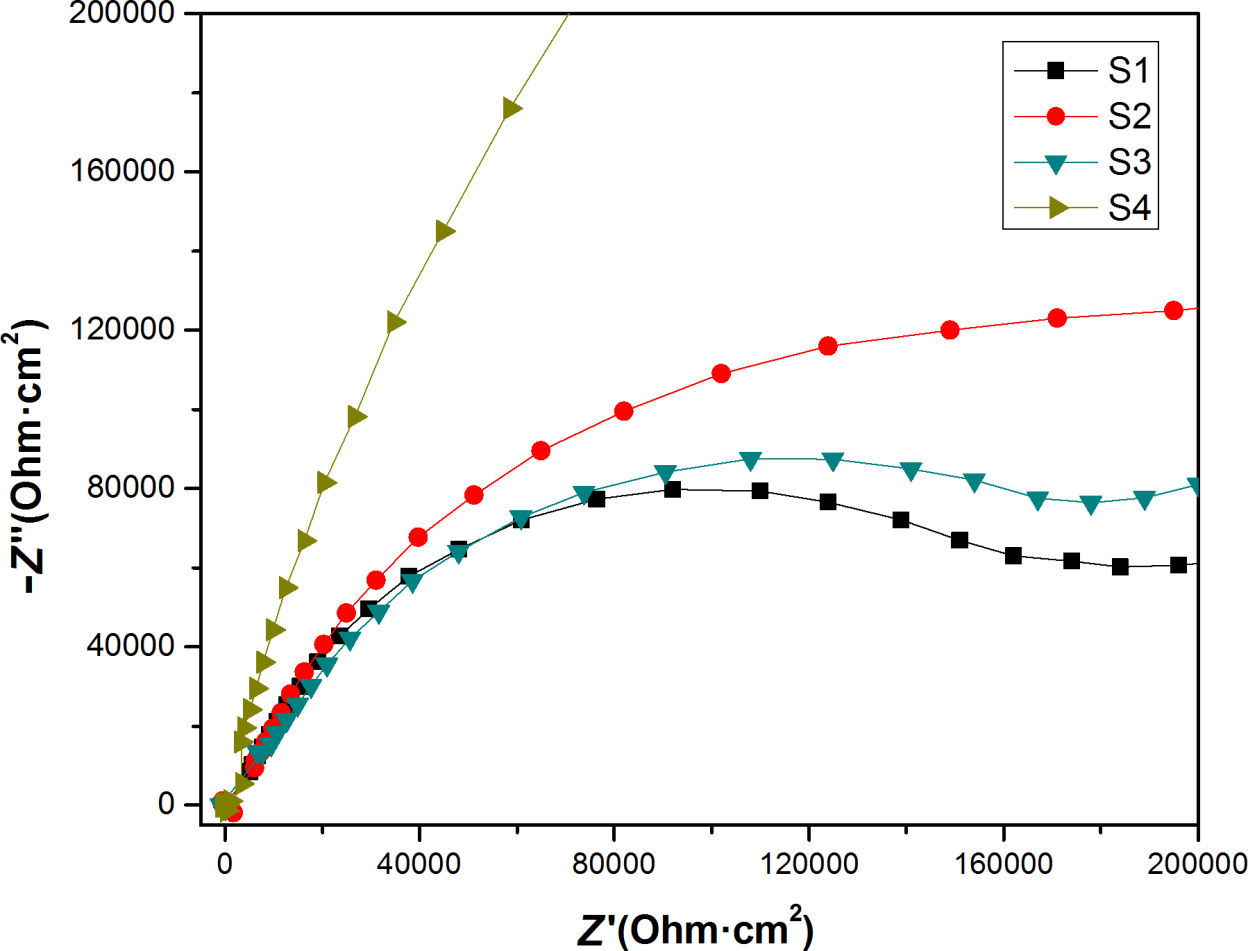
The zoomed portion of the high-frequency region of the Nyquist diagram for the various prepared samples.

The sealed AAO film is mainly composed of porous and resistant layers. A porous layer, an impedance layer, and an aluminum alloy substrate are present from top to bottom. While the electrical impedance test was performed to evaluate the performance of the porous layer of the AAO film, the low-frequency region corresponds to the performance of the impedance layer of the AAO film. During the electrical impedance measurements, the scanning begins in the high-frequency region and ends in the low-frequency region. From the Nyquist diagrams (Figure 8 and Figure 9), it can be noted that the data points remain roughly constant in the low-frequency region for all the prepared samples, but the capacitive loop varies from sample to sample. This reflects in an indirect way the different extents of corrosion in the porous layers. The capacitive loops as generated by the impedance layer exhibit the same trend. This demonstrates that the samples do not suffer from corrosion or corrode only to a very little extent. The Nyquist and Bode diagrams were fitted using the ZView software, and after a careful adjustment, equivalent circuit diagrams were obtained with the lowest fitting error (Supporting Information File 1, Figure S3).

As presented in Supporting Information File 1, Figure S3, the symbol *C* represents the capacitance, and CPE is the constant phase angle element that is used to refer to the electrical double-layer capacitance deviating from an ideal capacitance. The element with the capacitance *C* parallel to *R*1 designates the porous layer of the AAO film, while the other element with constant phase angle element parallel to *R*2 refers to the impedance layer in the AAO film. *R*2 represents the interfacial resistance of each blocking layer. The *R*2 values are presented for all the prepared samples in Table 5.

**Table 5:** Parameters obtained for each artificial circuit.

Sample^a^	Electrical impedance parameters				
	*C*1	*R*1 (Ω cm^2^)	*CPE*1-*T*	*CPE*1-*P*	*R*2 (Ω cm^2^)

S1	1.8593 × 10^−4^	86302	2.3295 × 10^−9^	3.0000	0.8407 × 10^5^
S2	4.4100 × 10^−3^	1.34700 × 10^−3^	2.2645 × 10^−12^	3.1870	1.1798 × 10^5^
S3	7.1404 × 10^−14^	22.15	1.4296 × 10^−5^	0.6978	3.5171 × 10^5^
S4	8.2153 × 10^−7^	54.38	4.8482 × 10^−6^	1.1470	5.1120 × 10^5^

^a^Sample S1: u(*C*1) = 3 × 10^-8^, u(*R*1) = 4, u(*CPE*1-*T*) = 2 × 10^−13^, u(*CPE*1-*P*) = 5 × 10^−4^, u(*R*2) = 60; Sample S2: u(*C*1) = 2 × 10^−7^, u(*R*1) = 9 × 10^−8^, u(*CPE*1-*T*) = 4 × 10^−16^, u(*CPE*1-*P*) = 3 × 10^−4^, u(*R*2) = 50; Sample S3: u(*C*1) = 5 × 10^−18^, u(*R*1) = 0.02, u(*CPE*1-*T*) = 7 × 10^−9^, u(*CPE*1-*P*) = 6 × 10^−4^, u(*R*2) = 50; Sample S4: u(*C*1) = 3 × 10^−11^, u(*R*1) = 0.04, u(*CPE*1-*T*) = 5 × 10^−10^, u(*CPE*1-*P*) = 2 × 10^−4^, u(*R*2) = 70.

The larger the value of CPE-P in the CPE is, the larger is the deviation of the artificial circuit capacitance from the theoretical capacitance, and the blocking layer of the sample S4 is closest to the theoretical capacitance among all the prepared samples. According to the results obtained via the electrical polarization and electrochemical measurements, the sample S3 possesses the best anti-corrosion performance.

## Conclusion

This paper has presented the preparation of a series of Cu–SiO_2_ NPs on a porous AAO film matrix by means of an alternating electrodeposition. As evidenced by SEM, XRD, EDS mapping, colorimetry, and electrochemical tests, both Cu and SiO_2_ NPs are uniformly dispersed in the porous AAO film matrices, albeit with a low content. There is strong XRD peak indexed to Al, but characteristic diffraction peaks assigned to Cu cannot be observed in the XRD patterns of all the prepared samples, revealing the low concentration of Cu NPs within the AAO film matrix beyond the detection limit of the XRD equipment. Only the sample S3 shows an obvious color change under different angles of incident light, i.e., the dark purple color changes to light purple when the incident light angle is changed from 0° to 30°. The electrochemical impedance and polarization test results reveal that the sample S3 exhibits the best anti-corrosion performance due to the optimal electrodeposition processing, yielding the highest quality of hole sealing in the porous AAO film.

## Supporting Information

File 1 Additional figures.

## References

[1] Boinovich L B, Modin E B, Sayfutdinova A R, Emelyanenko K A, Vasiliev A L, Emelyanenko A M (2017). ACS Nano.

[2] Wei H L, Elmer J W, DebRoy T (2017). Acta Mater.

[3] Chen K, Scales M, Kyriakides S (2018). Int J Mech Sci.

[4] Chang M, Wei H, Chen D, Hu H, Zhang Y, Ye X, Zeng K, Li D (2017). Nano Res Appl.

[5] Wei H, Hu H, Chang M, Zhang Y, Chen D, Wang M (2017). Ceram Int.

[6] Oddone V, Boerner B, Reich S (2017). Sci Technol Adv Mater.

[7] Mrad M, Ben Amor Y, Dhouibi L, Montemor M F (2018). Surf Coat Technol.

[8] Mugada K K, Adepu K (2018). J Manuf Processes.

[9] Edalati K, Horita Z, Valiev R Z (2018). Sci Rep.

[10] Fu J, Wang S, Wang K (2018). J Mater Sci.

[11] Martinez N, Kumar N, Mishra R S, Doherty K J (2018). J Mater Sci.

[12] Mrad M, Dhouibi L, Montemor M F (2018). Prog Org Coat.

[13] Seki Y, Ebihara K (2017). Surface-treated aluminum material and zinc-supplemented aluminum alloy. U.S. Patent.

[14] Gao W, Rigout M, Owens H (2016). Appl Surf Sci.

[15] Zhao X, Meng G, Xu Q, Han F, Huang Q (2010). Adv Mater (Weinheim, Ger).

[16] Zhang S-Y, Xu Q, Wang Z-J, Hao S-Z, Sun C-X, Ma W-J (2018). Surf Coat Technol.

[17] Liang C-J, Huang K-Y, Hung L-T, Su C-Y (2017). Surf Coat Technol.

[18] Wei H, Chen D, Hu H, Chang M, Ye X, Wang M (2017). RSC Adv.

[19] Tong L, Qi W, Wang M, Huang R, Su R, He Z (2016). Small.

[20] Kinoshita S, Yoshioka S, Miyazaki J (2008). Rep Prog Phys.

[21] Liu Y, Chang Y, Ling Z, Hu X, Li Y (2011). Electrochem Commun.

[22] Shi P, Wang F, Zhu J, Zhang B, Zhao T, Wang Y, Qin Y (2017). Ceram Int.

[23] Yang S M, Gu J J, Qi Y K, Wang J, Li X (2016). The study on structural color of porous anodic alumina thin film fabricated in phosphoric electrolyte. Electronics, Electrical Engineering and Information Science.

[24] Gao W, Rigout M, Owens H (2017). J Nanopart Res.

[25] Xu Q, Sun H-Y, Yang Y-H, Liu L-H, Li Z-Y (2011). Appl Surf Sci.

[26] Xu Q, Yang Y-H, Liu L-H, Gu J-J, Liu J-J, Li Z-Y, Sun H-Y (2011). J Electrochem Soc.

[27] Xu Q, Yang Y, Gu J, Li Z, Sun H (2012). Mater Lett.

[28] Yavuz G, Zille A, Seventekin N, Souto A P (2018). Carbohydr Polym.

[29] Jia Y, Zhang Y, Zhou Q, Fan Q, Shao J (2014). Thin Solid Films.

[30] Jia Y, Zhang Y, Liu G, Zhuang G, Fan Q, Shao J (2015). J Coat Technol Res.

[31] Jia Y R, Shao J Z, Fan Q G (2012). Adv Mater Res.

[32] Zhang Z, Zhang J, Hou X, Wu T, Sun H (2014). Thin Solid Films.

[33] Yuan W, Zhou N, Shi L, Zhang K-Q (2015). ACS Appl Mater Interfaces.

[34] Li Q, Zeng Q, Shi L, Zhang X, Zhang K-Q (2016). J Mater Chem C.

[35] Zeng Q, Ding C, Li Q, Yuan W, Peng Y, Hu J, Zhang K-Q (2017). RSC Adv.

[36] Wang Y, Han R, Qi L, Liu L, Sun H (2016). Appl Opt.

[37] Liu H, Sun H, Liu L, Hou X, Jia X (2015). Opt Mater.

