# Supplementary material for: An iridescent film of porous anodic aluminum oxide with alternatingly electrodeposited Cu and SiO2 nanoparticles
Source: Beilstein J Nanotechnol. 2019 Mar 19;10:735–45. doi: 10.3762/bjnano.10.73 (PMC6444397; doi:10.3762/bjnano.10.73)
Supplement: File 1 — Additional figures. [file Beilstein_J_Nanotechnol-10-735-s001.pdf]

BEILSTEIN JOURNAL OF NANOTECHNOLOGY

Supporting Information

for

An iridescent film of porous anodic aluminum oxide with alternatingly electrodeposited Cu and SiO_2 nanoparticles

Menglei Chang, Huawen Hu, Haiyan Quan, Hongyang Wei, Zhangyi Xiong, Jiacong Lu, Pin Luo, Yaoheng Liang, Jianzhen Ou and Dongchu Chen

Beilstein J. Nanotechnol. **2019,** *10,* 735–745. doi:10.3762/bjnano.10.73

Additional figures

License and Terms: This is a supporting information file under the terms of the Creative Commons Attribution License (http://creativecommons.org/licenses/by/4.0). Please note that the reuse, redistribution and reproduction in particular requires that the authors and source are credited.
The license is subject to the *Beilstein Journal of Nanotechnology* terms and conditions: (https://www.beilstein-journals.org/bjnano)

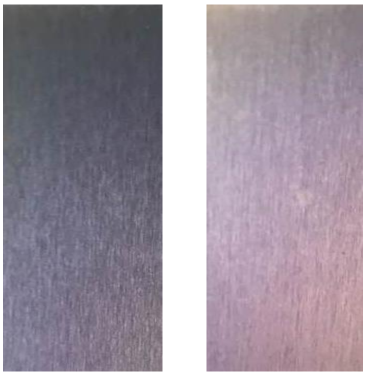

0° 30°

Figure S1: The color variation of the S3 sample under incident light at different angles (the sunlight perpendicular to the sample surface is designated to the incident light angle equal to 0°).

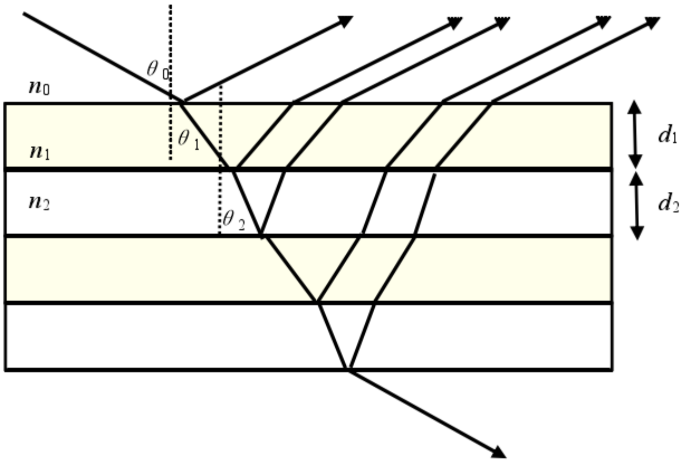

Figure S2: Schematic diagram of the interference of the multilayered films.

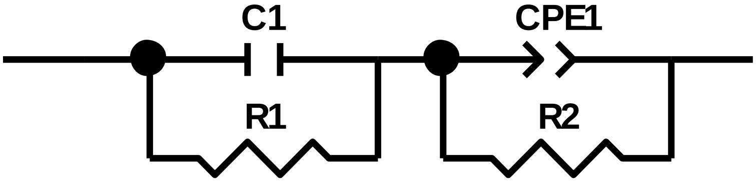

Figure S3: Equivalent circuit diagrams used in the calculation of impedance parameters.
